# Plant Group II LEA Proteins: Intrinsically Disordered Structure for Multiple Functions in Response to Environmental Stresses

**DOI:** 10.3390/biom11111662

**Published:** 2021-11-09

**Authors:** Mughair Abdul Aziz, Miloofer Sabeem, Sangeeta Kutty Mullath, Faical Brini, Khaled Masmoudi

**Affiliations:** 1 Integrative Agriculture Department, College of Agriculture and Veterinary Medicine, United Arab Emirates University, Al Ain 15551, United Arab Emirates; 201350444@uaeu.ac.ae (M.A.A.); 201990009@uaeu.ac.ae (M.S.); 2 Department of Vegetable Science, College of Agriculture, Kerala Agricultural University, Thrissur 680656, India; sangeeta.m@kau.in; 3 Biotechnology and Plant Improvement Laboratory, Centre of Biotechnology of Sfax (CBS), University of Sfax, B.P 1177, Sfax 3018, Tunisia; faical.brini@cbs.rnrt.tn

**Keywords:** abiotic stress, dehydrins, gene expression, group II LEA protein, hydrophilins

## Abstract

In response to various environmental stresses, plants have evolved a wide range of defense mechanisms, resulting in the overexpression of a series of stress-responsive genes. Among them, there is certain set of genes that encode for intrinsically disordered proteins (IDPs) that repair and protect the plants from damage caused by environmental stresses. Group II LEA (late embryogenesis abundant) proteins compose the most abundant and characterized group of IDPs; they accumulate in the late stages of seed development and are expressed in response to dehydration, salinity, low temperature, or abscisic acid (ABA) treatment. The physiological and biochemical characterization of group II LEA proteins has been carried out in a number of investigations because of their vital roles in protecting the integrity of biomolecules by preventing the crystallization of cellular components prior to multiple stresses. This review describes the distribution, structural architecture, and genomic diversification of group II LEA proteins, with some recent investigations on their regulation and molecular expression under various abiotic stresses. Novel aspects of group II LEA proteins in *Phoenix dactylifera* and in orthodox seeds are also presented. Genome-wide association studies (GWAS) indicated a ubiquitous distribution and expression of group II LEA genes in different plant cells. In vitro experimental evidence from biochemical assays has suggested that group II LEA proteins perform heterogenous functions in response to extreme stresses. Various investigations have indicated the participation of group II LEA proteins in the plant stress tolerance mechanism, spotlighting the molecular aspects of group II LEA genes and their potential role in biotechnological strategies to increase plants’ survival in adverse environments.

## 1. Introduction

A major part of the world is under the threat of water scarcity, salinity, and extreme temperature fluctuations. Plants face several forms of biotic and abiotic stresses in their natural habitats. These threats impose a drastic reduction in the survival and productivity of the crops. They account for half of the annual world plant production losses [1]. Various parts of plants, such as leaves, roots, and flowers, are very sensitive to small changes in the environment’s abiotic factors [2]. However, plants have incorporated well-developed stress-tolerant pathways and strategies that result in various kinds of modification at the genetic, biochemical, and physiological levels. It has been reported that the late embryogenesis abundant (LEA) proteins are crucial seed proteins, the accumulation of which acts as a functional adaptation to plants in acquiring tolerance against various abiotic stresses [3].

LEA proteins are largely hydrophilic proteins; they can prevent the damage caused by drastic environmental conditions [3]. They were found to contribute to numerous developmental processes and accumulate in relation to salinity, drought, freezing, and phytohormone and abscisic acid (ABA) treatments [4]. LEA proteins are divided into eight distinct groups based on their conserved motifs, amino acid sequences, and phylogenic relationships, such as LEA1, LEA2, LEA3, LEA4, LEA5, LEA6, dehydrin (DHN), and seed maturation protein (SMP) [5]. Among the LEA proteins, group II LEA proteins, or DHNs, are assumed to preserve macromolecules against injuries caused by drought, salinity, and freezing [6].

Group II LEA proteins are essential phytomolecules that accumulate mostly in the late phases of seed development and as a reaction to extreme external stresses in the vegetative tissues [3]. Among group II LEA proteins, DHNs constitute a distinct biochemical group known as LEA-D11 [5]. The expression profile of group II LEA genes governs the functioning of group II LEA proteins [7]. Group II LEA proteins were initially found in developing cotton (*Gossypium hirsutum*) embryos and are expressed in gymnosperms and angiosperms ubiquitously [8]. A positive association between the accumulation of group II LEA proteins and environmental stresses such as drought, heat, freezing, and salinity has been outlined in a number of studies [9]. However, in relation to contemporary genomics, these studies need to be reviewed and necessitate the generation of additional important structural, physicochemical, molecular, and functional characterization of group II LEA proteins.

The importance of the present review is to provide significant advances towards an in-depth understanding of the biological functions and activities of group II LEA proteins. The current review is necessary insofar as it provides a reference platform for revealing the group II LEA proteins’ role during plants’ adaptive responses to environmental stresses. Such a breakthrough will allow for speculation on using group II LEA genes or proteins in applications for several purposes in the field of biotechnology. Therefore, the current paper reviews the distribution and structural, architectural, and genomic aspects of group II LEA proteins’ diversification and molecular expression under various plant stresses using transgenic approaches. The paper also provides some insight on the *Phoenix dactylifera* group II LEA proteins and on the role of DHNs in orthodox seeds, with the aim of reinforcing their functional relevance under various environmental stresses.

## 2. Distribution of Group II LEA Proteins in Plants

The group II LEA proteins are found in both plants and animals but were initially characterized in cotton and wheat plants [10]. These proteins are involved in the maintenance of normal metabolism within higher plants, especially under the conditions of stress [4]. Group II LEA proteins were also identified in several other organisms such as algae, fungi, and cyanobacteria [11]. The group II LEA proteins are distributed within various plant tissues and at different developmental stages, indicating their important function throughout the plant growth cycle [8]. Group II LEA proteins accumulate highly in plant embryos during the late stages of seed development as an aid to embryo maturation under desiccation [3]. In plant vegetative tissues, group II LEA proteins are rarely detected and are limited to young parts of plants, especially those that exhibit excessive cell division and cell elongation, for example, at the root tips, in expanding stems, and in petioles [12]. However, once plants are under various stresses that lead to cellular dehydration, such as salinity, drought, temperature, and osmotic stress, group II LEA proteins accumulate into vegetative tissues at higher amounts than under normal conditions for the protection of different parts of the plant against the stress [11].

Some group II LEA proteins are found in mature seeds [8]. These distributions occur in Arabidopsis group II LEA genes, *RAB18*, and in *Zea mays*, *RAB17*. They are localized in all the segments of the embryo and endosperm of mature seeds [13] The *Pisum sativum* DHN gene, *DHN-COG*, accumulates in developing cotyledons during mid-to-late embryogenesis and in seedlings during dehydration stress [14]. It comprises about 2% of the proteins in mature cotyledons [13]. The carrot group II LEA gene, *ECP40*, is distributed in the zygotic embryos and endosperm of mature seeds [15]. Other group II LEA genes, such as *MAT1* and *MAT9*, were obtained from mature soybean seeds [16].

There are certain known group II LEA proteins that are distributed in vegetative tissues and in floral organs during the normal conditions of plant growth [12]. The Arabidopsis DHNs, *ERD14* and *ERD10*, were found to be distributed in the vascular tissues of leaves, stems, roots, and flowers [17], while the peach DHN, *PCA60*, was found in the shoot cells, including the tissues of the xylem and phloem, and in epidermal cortical cells [18]. Other group II LEA genes, such as wheat *WCOR410*, accumulated favorably in the vascular transport region of crowns, leaves, and roots of plants [18,19]. Some group II LEA proteins exhibit a localization to specific cell types, such as in guard cells, pollen sacs, and root meristematic cells [17]. Arabidopsis DHN, *RAB18*, accumulated specifically to the stomatal leaf guard cells [17].

Group II LEA protein content increases substantially under abiotic stress conditions and accumulates in different tissues than under standard plant conditions [11]. For instance, the Arabidopsis group II LEA genes, *ERD14* and *ERD10*, which were initially distributed at the tips of roots, in the stem tissues, and in the leaves and flowers of plants under favorable growth environments, appeared in the cells of all the tissues when plants were under cold stress [17]. *Solanum sogarandinum* and *Hordeum vulgare* DHNs, DHN24 and P-80, respectively, indicated a similar pattern of distribution under cold conditions [20]. The Arabidopsis group II LEA protein, LTI30, was not found in plants under normal growth conditions, but under cold conditions, it accumulated in the tissues of the roots, stems, leaves, flowers, and plant pollen sacs [17].

The wheat group II LEA protein, WCS120, was mostly confined in the vascular transport regions of crowns under cold stress but was not detected in the apical meristem of shoots or the mature xylem [21]. Another wheat DHN, WCOR410, was accumulated highly in the vascular transport area of leaves, crowns, and roots during plants’ cold acclimation [19]. The stomatal guard cell DHN in Arabidopsis, RAB18, was not induced under cold, but it was highly induced by ABA. The Arabidopsis ERD14 was also highly elevated in plants that were subjected to ABA and NaCl treatments [17]. The group II LEA proteins from *Craterostigma plantagineum*, DSP14 and DSP16, under normal conditions were identified in seeds, roots, and leaves, but under drought conditions, they were accumulated in all cells, preferentially in the embryonic cells of seeds and in the phloem of leaves [22]. Another group II LEA protein from *Lycopersicon esculentum*, TAS14 (YSK2), was barely accumulated under normal conditions but abundantly expressed in aerial parts and slightly in roots under the conditions of salinity stress [23].

Group II LEA proteins are also initiated in particular cells, including meristematic root cells, plasmodesmata, pollen sacs, guard cells, phloem, and nucelli [24]. Candat et al. examined the subcellular distribution of group II LEA proteins in Arabidopsis and found that with the exception of peroxisomes, all organelles contained one or more group II LEA protein in their cellular compartments as a plant protection assurance during stresses that lead to cellular dehydration [25]. In a number of plants, group II LEA proteins are accumulated in the plant cytoplasm, nucleus, mitochondria, chloroplast, and plasma membrane [24]. However, these proteins are more likely to occur in the cytoplasm or nucleus, and sometimes in both [26].

## 3. Sequence and Domain Architecture of Intrinsically Disordered Group II LEA Proteins

Group II LEA proteins are extremely hydrophilic and intrinsically disordered proteins (IDPs) that have a molecular mass ranging from 9 to 200 KDa [10]. Proteins that lack a well-defined three-dimensional fold are named as IDPs and may play a wide range of biological roles when they bind to their biological targets through folding (coupled folding and binding) [27]. IDPs are involved in many cellular functions, including regulation of cell division, transcription and translation, signal transduction, protein phosphorylation, storage of small molecules, chaperone action, transport, and regulation of the assembly or disassembly of large multiprotein complexes [10]. IDPs are depleted of hydrophobic amino acids (Val, Leu, Ile, Met, Phe, Trp and Tyr) and enriched with polar and charged amino acids (Gln, Ser, Pro, Glu, Lys, Gly and Ala) [27]. Consequently, they lack tertiary structure because they possess fewer hydrophobic residues to independently form a stable hydrophobic core [28]. Because of the low proportion of intramolecular hydrogen bonds between different amino acid residues, group II LEA proteins appear unstructured and share many features with other types of IDPs, such as their ability to change their conformation according to the changes in their ambient microenvironment [29]. The changes in protein conformation also result in changes in the protein function [28].

Group II LEA proteins can be distinguished from other LEA proteins by three conserved motifs [30]. They can be identified by a highly preserved 15 amino acid sequence motif that is lysine-rich (EKKGIMDKIKEKLPG), which is called the K-segment; a Y-segment located in the N terminus ([T/V]D[E/Q]YGNP); and an S-(serine-track repeats) motif [3]. The K-segment is considered the core segment of group II LEA proteins; it is an extensive segment and lays in one or more repeats, creating amphiphilic α-helixes at the C-terminal end of the proteins [26]. In relation to the arrangement and replication of these conserved motif sequences, group II LEA proteins are classified into five subcategories: Kn, KnS, YnKn, SKn, and YnSKn [30].

**Figure 1:**
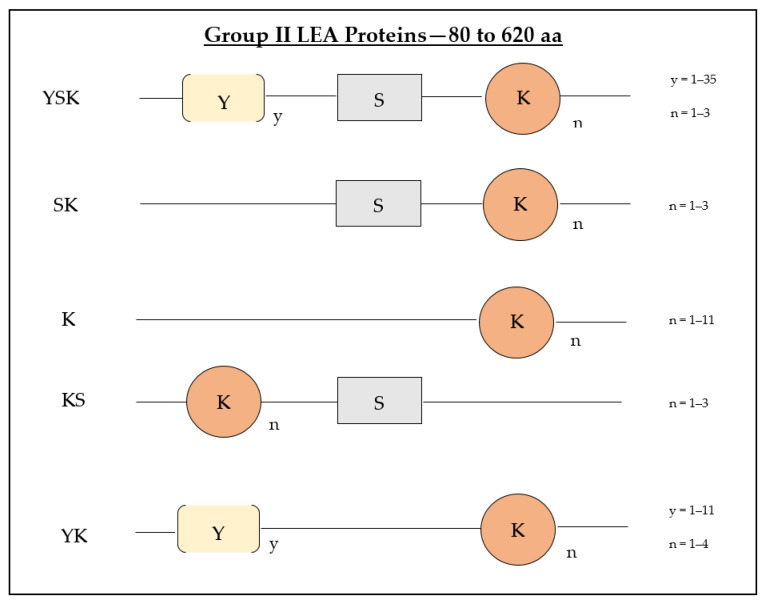
Schematic representation of the positions of repeated sequences that differentiate group II LEA protein subgroups. The blocks represent the arrangement of the motifs within the corresponding subgroups of group II LEA proteins. The numbers on the right indicate the tandem repeats of each motif in different subgroups. The size range of group II LEA proteins is indicated at the top as the number of amino acid (aa) residues.

The proteins that possess only a K-segment in their structural sequence belong to the K-subgroup of group II LEA proteins, and the SK-subgroup comprises those group II LEA proteins that contain an S-segment accompanied by a K-segment in their sequences (Figure 1) [3]. A new conserved segment of group II LEA proteins was found at the N-terminus (DRGLFDFLGKK), which was termed the F-segment [31]. It was recently determined in plants as an overlooked motif of group II LEA proteins that has potential functional properties of binding to membranes and other protein molecules [31].

The K-segments of group II LEA proteins interact with membranes and other proteins to modulate the proteins’ phase properties and conformational transitions [32]. The K-segment occurs in one to eleven copies within a chain of amino acids [33]. It was reported in a study that the wheat group II LEA protein, DHN-5, shielded the activities of β-glucosidase and lactate dehydrogenase (LDH) in vitro because of the presence of a K-segment in its amino acid sequences [34]. Furthermore, it was identified that in response to the application of sodium dodecyl sulfate (SDS), the *Citrus unshiu* K3S-type DHN, CuCOR19, formed an α-helix [35]. The Y-segment, representing a conserved segment, is usually present in one to thirty-five tandem copies and contains sequence similarities to the nucleotide binding sites of plants and bacterial chaperones [11]. Nevertheless, there has been no experimental documentation that the Y-segment binds to nucleotides [36]. The phosphorylation of the S-segment by protein kinase promotes group II LEA proteins’ interaction with particular peptide molecules and their transport to the nucleus as well as allowing them to bind to metal ions [37]. In addition to these conserved motifs, DHNs have the Φ-segment, which is less conserved and lies interspersed between K-segments [38].

Group II LEA proteins partially fold into α-helical structures under dehydration conditions [39]. This feature allows them to function as chaperones and prevent protein aggregation during abiotic stress [40,41]. The presence of the K-segment is responsible for the formation of amphipathic α-helices in the presence of helical inducers, which is relevant to DHNs’ function in response to drought-affiliated stresses [42]. Under stress conditions, α-helices can uphold membranes and proteins by protein–protein and protein–lipid interactions [43]. The structural properties of group II LEA proteins have been examined through a number of methods such as nuclear magnetic resonance (NMR) and circular dichroism (CD) [44].

### Evolution of the Structural Architecture of Group II LEA Proteins in Certain Plant Species

Group II LEA proteins’ evolution indicates the changes in the genetic sequence of these proteins, as throughout the process of evolution, there were gains of new group II LEA genes [45]. The changes in the gene sequence resulted in changes in protein molecules’ functional properties [6]. In a recent study, structural and phylogenetic analysis was conducted on 426 group II LEA gene sequences within 53 angiosperm and 3 gymnosperm genomes [45]. In angiosperms, the presence of all five architectures (Figure 1) was identified, whereas gymnosperms had only K and SK segments in their protein sequences [45]. This indicated that the ancestral group II LEA proteins that occurred in seed plants was a K or SK segment, and the group II LEA protein Y-segment first emerged in angiosperms. A high-level cleaving of the YSK segments of group II LEA proteins from either the K or SK segments could have been a possibility; however, after different duplication events, the lower-level structures of group II LEA proteins have evolved [46].

Malik et al. examined thirty-five angiosperm species that indicated the presence of at least one SK group II LEA protein [33]. Thirty-three species possessed no fewer than one YSK protein, while the other thirteen species had a minimum of one YK protein, fifteen species had K segments, and twenty-three species had at least one KS group II LEA protein [33]. The number of protein structures varied within plant species, with some plants having as many as nine group II LEA proteins with the same structure [33].

The evolution of group II LEA proteins has been examined in *Arabidopsis thaliana* [46], *Hordeum vulgare* [47], *Malus domestica* [48], *Brassica napus* [49], *Populus trichocarpa* [50], *Solanum tuberosum* [51] and wild relatives, and cultivated rice, Oryza [52]. These studies focused mainly on the evolution of group II LEA genes in a single species, indicating their evolution through gene duplication within the species [45]. However, examining the evolution of group II LEA proteins in the entire plant kingdom can provide larger insights into their origin and genomic functions in different species of plants.

## 4. Genomic Diversification of Group II LEA Proteins

The process of evolution has increased the genomic diversity of plant species [45]. This diversity has allowed plants to survive and adapt to different environmental conditions through the development and differentiation of specialized tissues [49]. Genomic diversity and mutations have been an important process for the diversity of proteins [22]. The genome sequence of group II LEA proteins has enabled the identification of different genes encoding for the corresponding proteins [33]. The genomic research in diverse group II LEA proteins has led to numerous advances in the understanding of their expression and function under different abiotic stresses [33]. The gene diversification of group II LEA proteins is thought to have occurred largely by duplication and functional divergence [45].

In genomic diversification analysis, identical genes include orthologs and paralogs, which are specific genes in different plants that originated from a single ancestral gene because of the process of replication [53]. Orthologs perform similarly in a number of plants, while on the other hand paralogs perform different functions and possess different specializations [54]. Paralog genes within similar plant species function more similarly than orthologs in different plant species that are present in the same plant diversification levels [53]. In addition to orthologs and paralogs, syntenic homologous genes or syntelogs are confined in identical regions of genomes that possess identical genomic bases in various plants, which evolved through a single ancestral gene [55]. Syntelogs are identified through the chains of synteny networks using different plant community identification methods [55]. Synteny networks indicate the locations of genes in similar regions of genomes of not closely related species [56].

In a study, a phylogenetic and microsynteny analyses of group II LEA proteins from 56 plant genomes revealed that the five structural subgroups of angiosperm group II LEA proteins can be assigned to three subgroups of orthologs, which was confirmed by the existence of the H-, F- or Y-segments [31]. Furthermore, it was found that in some plant species, group II LEA genes were paralogs that encoded for F-type proteins and were induced specifically by environmental stresses of salinity, heat, and drought [31]. This indicates that the ancient synteny diversification of group II LEA proteins in flowering plants caused protein sequence and biochemical alterations. The differences in the expression patterns of group II LEA genes patterns may be associated with their functional peculiarity [57]. However, additional experimental evidence is required to examine these changes.

In another study, a large genomic analysis of LEA proteins was performed across 60 genomes of different plant species [46]. The analysis identified eight multigene families for the eight different groups of LEA proteins. It was found that around 4836 differential genes were distributed in the LEA protein genome, among which group II LEA genes were profusely occurring with 3,126 genes that were spotted in the bryophyte clade, *Physcomitrella patens*, in angiosperms and lower plant genomes [46]. The different copy numbers of group II LEA genes among different taxa indicate the individualistic loss of these genes and the replication of these genes in separate single plant genomes [31]. The replication of group II LEA genes is correlated to the tolerant lifestyle of different plant species, indicating that the evolution of group II LEA genes contributed to water stress adaptation in plants [45].

In order to investigate the localization of group II LEA proteins in angiosperms, Artur et al. analyzed the content of Glycine (Gly) and the GRAVY index of group II LEA proteins within the two angiosperm communities [58]. Although the hydrophilic property of group II LEA proteins was present in both the communities, community 1, in comparison to community 2, possessed proteins that contained a different composition of Gly/GRAVY. The protein molecules in community 2 possessed a more similar composition of Gly/GRAVY [58]. These detections indicated that group II LEA proteins do not construct separate synteny communities; rather, they possess diversified biochemical properties, which originated from different plant genomes [59].

### Genome-Wide Association Studies (GWAS) of Group II LEA Proteins

GWAS are used for investigating various genes and their multiple or complex traits in relation to different stresses [60]. The group II LEA genes in plant genomes exhibits diversity in size, sequence and complexity that is equally related to the diversity of their form and function in plants [61]. GWAS of group II LEA proteins in different plants have identified novel group II LEA genes that were responsible for tolerance to biotic and abiotic stresses [60]. The outcomes of these investigations on different plant stresses will be essential for the selection of genes and designing of future crops.

In *Populus trichocarpa*, 88 LEA genes were identified on the basis of a genome-wide search; these genes had fewer introns and contained more cis-regulatory elements in their promoters related to tolerance to abiotic stresses [61]. Among these genes, the group II LEA genes had the maximal number of LEA genes, accounting for 60 of them. A comparative genomic analysis revealed that these genes were conserved and homologous to related genes in other plant species such as *Arabidopsis thaliana*, *Eucalyptus robusta*, and *Solanum lycopersicum* [61]. In addition to this, the pepper genome was analyzed through GWAS, which indicated that seven candidate group II LEA genes were mapped on pepper chromosomes, with four genes on chromosome 2, one on chromosome 4, and the last two on chromosome 8 [62]. Also, in *Oryza sativa ssp*. *japonica*, a total of 65 group II LEA genes were identified using GWAS on 11 Oryza plants [63]. Moreover, a GWAS carried out on apple plants revealed 12 group II LEA genes that were located on various chromosomes [48]. The putative proteins obtained from those genes contained a K domain typical of group II LEA proteins [48].

In *Physcomitrella Patens*, seven group II LEA genes were identified through GWAS [64]. The sequence alignment analysis of the putative proteins from these genes indicated a typical K domain similar to the apples [64]. The *Physcomitrella patens* group II genes were expressed in all vegetative tissues, while in young leaves and shoot tips, some of these genes were not expressed. Furthermore, a GWAS resulted in the identification of seven group II LEA genes in *Zea mays* and *Setaria italica* as well as five in *Sorghum bicolor* that were classified into the five subgroups of group II LEA proteins [65]. The group II LEA genes of Sorghum displayed one ortholog with *Oryza sativa* and *Zea mays* and three with *Setaria italica*, whereas *Sorghum bicolor* group II LEA genes encoded for ordered proteins that possessed many phosphorylation sites [65]. In another GWAS, seven group II LEA genes were identified in *Actinidia chinensis* that belonged to putative proteins of YSK and SK groups [66]. These genes were highly expressed in stems, leaves, roots, and fruits. During the leaf growth, the expression levels of some of these genes were downregulated, and during fruit growth, they were upregulated [66]. These findings suggested that group II LEA genes also play a role in the regulation of leaf or fruit development [66]. However, under the different abiotic stresses of salinity, drought, and high and low temperatures, the transcription levels of these genes were significantly increased.

With the advent of GWAS, four group II LEA genes were identified in both *Vitis vinifera* and *Vitis yeshanensis* [67]. The two species had high sequence similarity, but between the group II LEA genes, there was little homology. All four group II LEA proteins possessed hydrophilicity but varied in their isoelectric points, kinase selectivity, numbers of functional motifs, and expression profiles. Some of these genes were not expressed in vegetative tissues under normal growth conditions but were highly expressed under abiotic stresses [67]. In *Picea glauca*, 41 group II LEA coding genes were found, and a phylogenetic reconstruction indicated that these genes underwent an expansion in conifers, with sporadic resurgence of specific amino acid sequence motifs, and that duplication of these genes gave rise to a clade specific to the Pinaceae [68].

A comparative genomics study was performed in four model Brachypodium grass species’ (*Brachypodium distachyon*, *Brachypodium stacei*, *Brachypodium hybridum* and *Brachypodium sylvaticum*) group II LEA genes [69]. Genomic sequence analysis detected ten group II LEA genes across the Brachypodium species’ 47 LEA genes. The YSK2 structure of group II LEA protein was most commonly encoded by *Bdhn* genes. Brachypodium genes were laid across various chromosomes, and most commonly on the same chromosomes: three and four of *Brachypodium distachyon*, four and five of *Brachypodium stacei* and four of *Brachypodium sylvaticum*. It was indicated that tandem and segmental replication incidence occurred for four *Bdhn* genes. These genes had three upstream cis-regulatory motifs. Some expression of these genes was found in mature leaves, particularly under the stress of drought. These genes were similar to wheat orthologs that were also highly expressed under drought stress. The expression of Brachypodium group II LEA genes corresponded remarkably to drought-responsive phenotypic traits such as the content of water, proline, and carbon within the plant and its biomass [69].

## 5. Group II LEA Gene Expression and Regulation Patterns under Abiotic Stresses

The expression of group II LEA proteins or DHNs can be triggered by numerous abiotic factors such as heat, salinity, and drought as well as by phytohormones such as ABA [4]. Hence, group II LEA proteins are also termed as responsive to abscisic acid (RAB) proteins [36]. The overexpression of DHNs in certain investigations has been reported to enhance tolerance towards abiotic stresses [6]. The major functions of DHNs detected when overexpressed are their significant participation in stabilizing enzymes, membranes, proteins, and cell nucleotides under abiotic stresses [70,71].

### 5.1. Expression of Group II LEA Genes under Salinity Stress

The salt stress tolerance mechanism within plants has been substantially studied and specified in a number of plants; it involves both ABA-dependent and ABA-independent signaling pathways [72]. Salinity stress disrupts plant growth and development through moisture and cytotoxicity stress, which occurs because of excessive uptake of ions such as sodium (Na^+^) and chloride (Cl^−^) and results in nutritional imbalances and eventually cell damage [73].

Salinity stress triggered the overexpression of group II LEA proteins obtained from Durum wheat (DHN-5) in transgenic Arabidopsis, which enhanced its tolerance towards salinity through modulation of the interaction at both the transcriptional and protein levels [74]. In banana, an SK(3)-type DHN gene, *Musa DHN-1*, was identified, and its overexpression led to improved salt tolerance in transgenic banana, as confirmed via expression profiling in both leaves and roots [75].In addition, heterologous expression of two DHNs from *Physcomitrella Patens*, *PpDHNA* and *PpDHNC* in *Arabidopsis thaliana*, revealed stronger tolerance to salinity than wild-type and empty-vector control lines [76]. Another study revealed that transgenic Arabidopsis expressing *CaDHN4,* a DHN gene from pepper (*Capsicum annuum* L.) leaves, in comparison to wild type plants, displayed higher seed germination rate and postgermination primary root growth under salt stress [77].

Furthermore, the application of methyl jasmonate (MeJA) has been shown to be effective, especially under salinity stress, at improving plant tolerance, resulting in a two-fold increase in the level of DHNs under salinity and enhancing the protective properties of the cell wall through lignin deposition acceleration in wheat seedling roots [78]. Moreover, the overexpression of *Hevea brasiliensis* DHNs, *HbDHNs*, exhibited a significant salinity tolerance increase in *Arabidopsis thaliana* [79]. In another study, the phylogenetic aspects of the *Avicennia officinalis* DHN 1 gene, *AoDHN1*, were analyzed, showing that it belongs to the group II LEA genes and revealing transcript upregulation in response to salt treatment [80]. In many contexts, the behavior of DHN genes in protease activity has also been studied via experiment [76]. The results have indicated that DHNs are vital for plant stress responses to salinity and can be exploited to develop more salt-resilient germplasm that boosts their growth and development.

### 5.2. Expression of Group II LEA Genes under Drought Stress

Drought is a major environmental stress limiting food production around the world through the growth and yield inhibition of plants under extreme drought periods [73]. Plant cells react to drought stress through the accumulation of osmotically active compounds such as hydrophilic DHNs [81]. A positive correlation has been revealed between the build-up of group II LEA gene transcripts or proteins and plant drought stress adaption in a number of physiological studies focusing on plant responses towards stress [82]. It was found that drought-tolerant cultivars or genotypes had higher content of DHN transcripts or proteins than less tolerant cultivars [82]. However, because of the complicated mechanisms of plant stress tolerance, the relationship between the aggregation of group II LEA proteins or gene transcripts and plant stress resistance is not always clear [83].

Drought stress can instigate secondary stresses in the form of oxidative and osmotic stress [73]. In vivo studies indicated DHNs’ role in protecting enzymatic activities from inactivation under in vitro partial water limitation, which suggested one of its functional properties under drought [84]. A comparative analysis conducted on drought-resistant wheat cultivars (Omskaya35—O35 and Salavat Yulaev—SYu) for their physiological and biochemical characterization showed that the loss of water resulted in the accumulation of DHNs, specifically low-molecular-weight DHNs, which were 2.5 times higher in abundance in the O35 cultivar than in the SYu cultivar [85]. Furthermore, the overexpression of the *Caragana korshinskii* (Fabaceae) group II LEA gene, *CkLEA2-3*, in *Arabidopsis thaliana*, led to greater tolerance to drought stress [79]. Since drought triggers rapid production of phytohormone ABA, which in turn induces expression of RAB stress-related genes, expression of DHN genes occurs under these conditions of dehydration as its regulation is controlled by both ABA-dependent and ABA-independent signaling pathways [86]. Moreover, the ubiquity of expanded helical structures and disordered configurations in DHNs is compatible with its role of conserving adequate moisture within the cellular compartments during dehydration stress [87].

It has been shown that several transcription factors and regulators also play an important role in the regulation of drought-resistant proteins in response to reduction in cell water content [88]. A positive regulator of drought response, the *Medicago truncatula MtCAS31* (cold-acclimation specific 31) DHN, aided in autophagic degradation [89]. Its role in the autophagic degradation pathway and expression under the stress of drought was indicated through a GFP cleavage assay and with an autophagy-specific inhibitor treatment [89]. The wheat DHN gene, *Wdhn13*, from *Triticum boeoticum* exhibited a high expression level in comparison to the levels in another tolerant cultivar (Sirvan) and other wild species under drought conditions [90]. In wheat species, there was a remarkable correlation of the drought tolerance at the gene-transcript level and the properties of the antioxidant enzymes, such as ascorbate peroxidase, superoxide dismutase, and glutathione peroxidase, of the same species [90]. The regulatory mechanism of differentially expressed genes (DEGs) was identified in rice under drought stress conditions [91].

It was reported that in the regulation of the DHN gene cluster, a reciprocity between histone *H3K4me3* modification and transcription factor *OsbZIP23* enhanced tolerance to dehydration [92]. It was found that a DHN gene from *Solanum habrochaites*, *ShDHN*, was expressed at its maximum level of 12-fold under drought stress within 6 h [93]. Furthermore, another DHN gene from *Saussurea involucrata*, *SiDhn2*, increased to 12-fold expression within 3 h of drought [93,94]. However, a DHN gene from wheat, WZY2, displayed a lower reaction to moisture loss for the highest expression level at 24 h of drought condition [95]. As a result, it can be stated that the time intervals of different DHN genes’ reactions towards drought stress stages differ.

There are dehydration-responsive elements (DREs) in some DHNs (A/GCCGAC motifs) accompanied or not by ABA-responsive cis-elements, ABRE motifs [96]. The presence of a DRE motif is considered as a key domain for the response of DHN genes towards drought stress in the ABA-independent pathway [96]. Some studies have indicated the binding capability of transcription factors DREB1 and DREB2 to the DRE element in Arabidopsis rd29A for the mitigation of the drought stress [97].

### 5.3. Expression of Group II LEA Genes under Temperature Stress

Temperature stress occurs because of fluctuations in the air temperature in a plant’s environment, which determines the plant’s phenology [98]. Many developmental processes are impacted by seasonal changes in temperature [99]. Some processes that are sensitive to temperature include plant flowering and seed germination [99]. However, certain variations in temperature thresholds restrict the geographical distribution and productivity of crops [98]. Changes in temperature build fluid imbalances in the plant cell membrane, causing metabolic disturbances [100]. Temperature stress can be due either to high heat or cold in the environment [86]. Heat stress is a complex phenomenon that denatures and aggregates protein molecules, while cold stress results in the formation of ice crystals in extracellular spaces and diminishes the portion of liquid water in the cells [101].

It was found that a DHN gene from *Cucumis sativus*, CSLEA11, and wheat WZY2 proteins provided protection to LDH enzyme activity in recombinant *Escherichia coli* under heat stress [102,103]. The presence of certain heat stress elements (HSEs) in the promoters of wheat DHN genes, *TaDHN1* and *TaDHN3*, was involved in the wheat’s response to heat stress [104]. However, the presence of HSEs in the promoters of other DHNs remains obscure. The expression of group II LEA proteins enhanced protection against low-temperature stresses in various plant species [75]. In Arabidopsis, overexpression of *AtDREB1A* or *AtDREB2A* induced cold stress-related genes such as *rd29A* and *COR47* [105]. It was reported that a number of DHNs induced by cold stress were identified in Arabidopsis, soybean, and rice based on a certain microarray analysis [106]. In another study, overexpression of the *Prunus mume* DHN gene, *PmLEAS*, enhanced the tolerance towards cold in tobacco and *Escherichia coli* [107]. Examination of the purified maize (*Zea mays*) G50 DHN indicated its potent cryoprotective activity under cold stress, specifically with the presence of compatible solutes [32].

Furthermore, the detrimental impacts of freezing and ionic stress were improved through the overexpression of a group II LEA protein from tomato (*Lycopersicon esculentum*) in yeast [108]. Moreover, osmotic and cold stress were stimulated by treatment with ABA, which is compatible with the certainty that the ABA-responsive element was initially reported in group II LEA gene from *Oryza sativa* [109]. As a result, group II LEA genes were overexpressed under cold stress because of the presence of ABA-responsive elements, and its response towards stress Was mediated by ABA [17].

The cosegregation of the DHN gene under the chilling stress indicated its role in stress tolerance during seedling emergence in cowpea [110]. It was found that the same stresses did not result in upregulation of DHNs; rather there was an increase in DHN mRNA levels in response to different abiotic stressors [111]. Some studies have indicated that DHN gene promoters possessing DREs did not react to cold or drought stresses [96]. This shows a complex pathway of cold stress regulation in DHN genes, which requires extensive examination to understand.

### 5.4. Expression of Group II LEA Genes under Osmotic Stress

Plants’ exposure to different environmental stress conditions of salinity, drought, and low temperature results in osmotic stress, because of which the growth and productivity of plants decline [100]. Osmotic stress in plants lowers the chemical potential of water external to the cell and causes the movement of water out of the plasma membrane, resulting in dehydration [73]. Plants respond to osmotic stress through the accumulation of ABA, which induces the production of group II LEA proteins [112].

In a study conducted on *Physcomitrella patens*, a knockout DHN mutant generated using homologous recombination showed minimal growth [113]. However, transgenic Arabidopsis plants expressing two DHN genes from *Physcomitrella patens*, *PpDHNA* and *PpDHNB*, showed improved root growth under osmotic stress [114]. High concentration of salt causes cellular osmoticstress, and the tolerance mechanism involves conservation of the equilibrium of cellular ions, osmotic acclimatization, and reactive oxygen species (ROS) scavenging [115]. The osmotic pressure in turn increases the concentration of Ca^2+^ and inositol 1, 4, 5 triphosphate (IP3) in the cytosol [116]. Ca^2+^ and IP3 act as secondary messengers, which activate the mitogen-activated protein kinase (MAPK) cascades for the regulation of phosphorylation of different transcription factors such as CBF/DREB, ABF, Bzip, Myc/MYB, and NAC (NAM, ATAF, CUC) factors [117].

A *Pennisetum glaucum* DHN gene, *PgDHN*, in transformed *E. coli* cells led to enhanced tolerance and generated a higher growth rate during salinity stress at a concentration of 750 mM and during osmotic stress in comparison to control *E. coli* cells [118]. Furthermore, the heterologous expression of *PgDHN* in transgenic yeast led to improved tolerance to a number of abiotic stresses [118]. In another study, transgenic *Arabidopsis thaliana* lines that expressed different forms of DHN-5 gene from *Triticum durum* Desf., with or without the K-segments were generated [119]. The results indicated that the constructs possessing only one or two K-segments improved the tolerance of the *Arabidopsis thaliana* seedlings to multiple stresses and were found identical to the full-length DHN-5 gene. Moreover, in comparison with the YS form and the wild type, the transgenic plants with K-segment constructs conserved higher catalase and peroxide dismutase enzymatic activity and maintained lower levels of malondialdehyde and H_2_O_2_ [119]. Moreover, in a study, the overexpression of a *Caragana korshinskii* (Fabaceae) group II LEA gene, *CkLEA2-3*, in *Arabidopsis thaliana*, led to enhanced protection to osmotic stress under the seed germination stage [120].

Two DHN genes from *Agapanthus praecox*, *ApY2SK2* and *ApSK3*, displayed essential protective effects during complicated stresses [121]. The overexpression of *ApY2SK2* and *ApSK3* in *Arabidopsis thaliana* led to reduction in damage to the plasma membrane and ROS levels and caused higher antioxidant activity and photosynthesis capability during osmotic stresses in comparison to the wild-type species [121]. In a study, a YSK2-type DHN from *Sorghum bicolor*, *SbDhn1*, generated a high level of transcript accumulation when exposed to osmotic stress. The overexpression of *SbDhn1* in transgenic tobacco lines led to enhanced osmotic stress tolerance, as examined through reduced membrane damage and lower levels of malonyl dialdehyde (MDA) [122]. Furthermore, it was found that the transformation of Arabidopsis with three DHN genes from *Ammopiptanthus mongolicus*, *AmDHN132*, *AmDHN154*, and *AmDHN200*, enhanced its tolerance towards cold, salinity, and osmotic stress [123]. However, the highest tolerance was indicated by the *AmDHN132* gene [123]. Another study related to an intrinsically disordered Arabidopsis DHN gene, *ERD14*, revealed that its overexpression with increasing levels of H_2_O_2_ under osmotic stress protected the enzymes through its chaperone-like properties [124]. Moreover, through the overexpression of a *Capsicum annuum* DHN gene, *CaDHN5*, in Arabidopsis improved its tolerance towards salt and osmotic stresses in comparison to wild type [125]. Through genome-wide identification, expression profiling, and functional analysis, some recent advancements in the functioning of DHNs against different abiotic stresses in various plants are highlighted in the Table 1.

## 6. Group II LEA Protein or DHN Responses to Biotic Stresses

The role of group II LEA proteins in countering abiotic stresses is widely known [4]. However, their role towards biotic stresses has been poorly studied. DHNs respond to wounds caused by biotic stresses through exogenous hormones that are essential in pathogen defense signaling and disease resistance, such as ABA, ethylene, jasmonic acid and salicylic acid [41]. Thus, the activity of DHNs in plant defense during pathogen attacks remains to be examined [44].

Few studies have displayed an association between the expression of DHNs and plant responses towards fungal infection, both individually and in occurrence with abiotic stress [67]. In a study, a DHN-like gene, *BcDh2*, extracted from *Boea crassifolia* was highly expressed under salinity, drought, and ABA treatment, while its accumulation was lower in response to signals of wounding, such as the release of methyl jasmonate, and during low concentration of salicylic acid [153]. Furthermore, the overexpression of the Arabidopsis DHN gene *AdDHN1* increased the susceptibility of transgenic Arabidopsis lines to *Meloidogyne incognita*, a biotrophic plant pathogen with a varied host range and complex colonization of the host plant [154]. In another study, overexpression of an Arabidopsis DHN gene, *SAG21*, led to increased resistance towards *Botrytis cinerea* and *Pseudomonas syringae*, a virulent bacterial pathogen [155].

## 7. Accumulation of Group II LEA Proteins in *Phoenix dactylifera*

*Phoenix dactylifera* is a crucial plant in arid and semiarid regions [156]. It is a woody and extremophile plant that thrives under high heat, drought, and salinity [157]. Dramatic changes in the abiotic factors of the arid regions have resulted in a decline in its production [158]. Many studies have characterized the consequences of abiotic stresses on the growth and physiology of date palm and focused on its tolerance mechanism for the functional characterization of abiotic stress responsive genes [159,160]. As a desert plant with a native tolerance to wide range of abiotic stresses, the date palm may act as a treasure store of novel genetic resources that can be exploited for abiotic stress tolerance [160]. Although a number of physiological, molecular, and biochemical analyses of stress-related genes in date palms have been documented, research on the functional properties of date palm group II LEA genes is still scarce [156,157,161]. The lack of analyses of DHNs in date palm has been associated to its many varieties, of which only a few are dominant and actively cultivated [162].

In a recent study, date palm leaves were treated with ABA to mimic the effects of drought stress [163]. The study reported a DEG analysis between ABA-treatment and control conditions and showed an extensive overlap in DEGs in date palm and drought stress-responsive genes in Arabidopsis. For instance, the authors observed an extensive accumulation of LEA proteins that were known to play a role in Arabidopsis abiotic stress responses. The accumulation of the *P. dactylifera* LEA proteins indicated their role in date palms’ tolerance to a wide range of abiotic stresses.

Transcriptional regulation of the LEA family was obscurely analyzed in date palm species. However, a whole genome sequencing of Khalas variety of date palm was carried out by Al-Mssallem et al. [164], a genetic map was constructed by Mathew et al. [165], and a computational characterization of a group of conserved miRNAs was conducted by Xiao et al. [166] based on the genome of the Khalas variety. The investigations from the whole genome sequencing of date palm Khalas variety broadened the identification of LEA genesand divided them into eight groups and eighty-four gene members within the taxa [164]. The authors indicated an abundance of DHNs or group II LEA genes in the date palm genome assembly, which included sixty-two variants of group II LEA genes. According to the transcriptomic data, Al-Mssallem et al. [164] showed a complex ABA-induced expression profile in different organs and developmental stages of date palm.

There is ubiquitous occurrence of group II LEA proteins in date palm [163]. The evolution of DHNs in date palm is due to the multiple abiotic stresses present in its natural habitat, and the abundance of group II LEA proteins indicates a possible role in date palms’ stress tolerance that needs further investigations. Novel interrogations of date palm group II LEA genes may expand germplasm resources. Through genome engineering and genetic manipulations via CRISPR-Cas9, date palm varieties with group II LEA proteins will be produced to ameliorate the agricultural production of date palms [164].

## 8. DHNs Relation in Storage and Conservation of Orthodox and Recalcitrant Seeds

In seed physiology, DHNs or group II LEA proteins are considered to be responsible for the persistence and longevity of seeds [167]. Plant seeds are of special interest for investigating the proteins from the group II LEA family, since they are relatively abundant during seed maturation stages and in response to any external stimulus causing dehydration to the seeds [3]. Seeds are classified as recalcitrant or orthodox based on their storage behaviors [168].

Recalcitrant seeds do not go through maturation drying and drop with a relatively high content of moisture [169]. Seed recalcitrance is a major issue for the natural production of plant species that causes a serious problem in seed conservation and storage [170]. In recalcitrant seeds, a positive correlation was found between the seed moisture content and the germination rate [169]. These seeds cannot be maintained and stored in conventional freezers due to their low survivability after drying and freezing at −20 °C. The absence of resistance in recalcitrant seed drying was attributed to the lack of DHNs [171].

Orthodox seeds, on the other hand, go through maturation drying and are dropped from plants at a low content of moisture [172]. These seeds have the potential to be dried to an internal seed water content of less than 12% and can be maintained, stored, and survived at freezing temperatures [172]. DHNs are synthesized in orthodox seeds, which are accumulated during the final stages of maturation and during seed desiccation [173]. It has been suggested that, in orthodox seeds, DHNs favor their tolerance towards moisture loss and osmotic stress during the stage of seed maturation [174].

**Figure 2:**
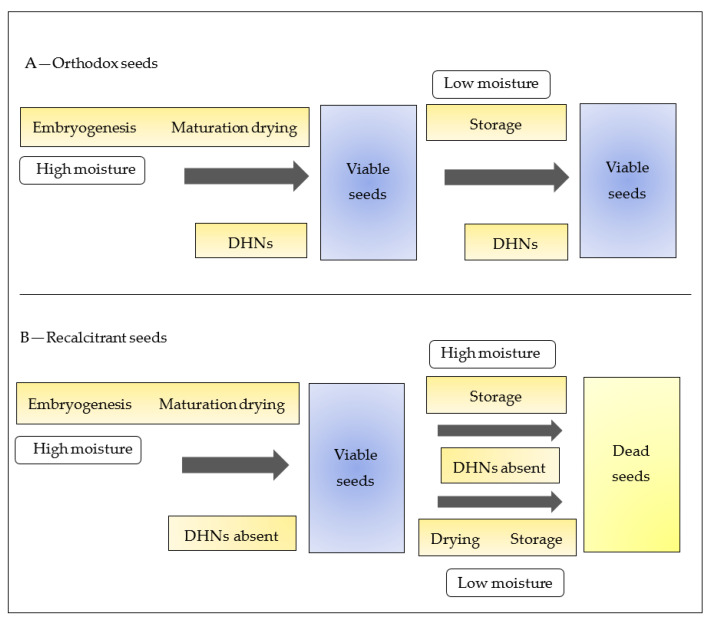
DHN expression during maturation drying (**A**—orthodox seeds, **B**—recalcitrant seeds). (**A**) Orthodox seeds synthesize DHNs as a response to maturation drying and to storage under low moisture conditions and retain their viability after seed storage. (**B**) Recalcitrant seeds lack DHNs during maturation drying and storage under low and high moisture conditions; therefore, they become unviable after drying and seed storage.

There are various protective mechanisms that are induced during maturation drying, such as the production of DHNs [175]. The consequence of the production of DHNs is obvious when contrasting the DHN expression in orthodox and recalcitrant seeds [173]. In orthodox seeds, as a response to the maturation drying, DHNs are synthesized, whereas recalcitrant seeds do not synthesize DHNs because of the absence of any maturation drying [172]. It is because of DHNs that orthodox seeds retain their viability during storage (Figure 2A), whereas recalcitrant seeds become unviable because of the absence of DHNs during maturation drying and storage conditions. (Figure 2B).

In a study that investigated the long-term storage and conservation of *Vateria indica* seeds, an interaction was displayed between moisture content and ABA levels during seed desiccation, which may have an influence on DHNs and their induction [170]. In another study, recalcitrant *Citrus limon* seeds did not display any reaction to desiccation tolerance when three group II LEA genes were downregulated upon paclo butrazol (PAC) treatment [176]. Therefore, it is of crucial importance to understand the molecular mechanism and functional properties of DHNs in plant seeds, as they influence seed physiology and acquisition of desiccation tolerance and thus protect the genetic resources of plants.

## 9. Group II LEA Proteins’ Functional Heterogeneity

Plants have initiated multipathways, which have enabled various responses to different environmental stresses [6]. Group II LEA proteins are regarded as stress proteins that play an essential role in plants’ protection under stress [4]. In vitro experiments have shown the ability of group II LEA proteins to protect enzymatic activities from damage caused by abiotic stresses [177]. Although multiple studies have been undertaken to understand their function under environmental stresses, their molecular role still remains obscure. In this review, novel insights related to DHNs were generated that characterized their functional properties under the stress environment, as it is crucial to have an extensive understanding about their biochemical, physiological, and biological functions in plant stress management. A number of transgenic approaches have indicated that overexpression of group II LEA proteins in a wide range of plant species improves abiotic stress resistance [9].

### 9.1. Biomolecule Preservation

**Figure 3:**
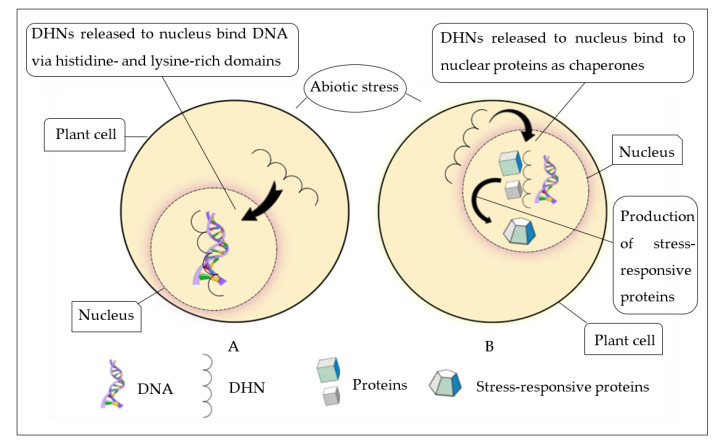
Role of DHNs inside the nucleus of a cell (**A**—binds to DNA, **B**—binds to other protein molecules). (**A**) Nuclear-localized DHN under stress conditions binds to DNA because of the presence of DNA-binding domains (histidine and lysine), which may repair or protect the DNA from damage caused by abiotic stresses. (**B**) DHNs shield protein molecules through protein–protein interaction as chaperones in the nucleus and facilitate the production of stress-responsive proteins.

One of the key functions of group II LEA proteins is their ability to protect biomolecules under stress conditions [4]. Group II LEA proteins shield partially denatured proteins and prevent the occurrence of protein–protein aggregation under limited water and cold conditions [32]. The property of protein antiaggregation activity of DHNs expands to the protection of complicated protein molecules [178]. The nuclear localization of DHNs indicates two possible functional characteristics of DHNs [6]. Group II LEA proteins mainly act as chaperones and bind to DNA (Figure 3A) and other protein biomolecules by shielding them and thereby preserve the functions of these proteins during the stress (Figure 3B).

A study conducted by Boddington and Graether et al. [179] revealed that a nuclear-confined group II LEA gene from *Vitis riparia Michx.*, *VrDHN1*, has the ability to bind to DNA and protect it from immoderate ROS such as H_2_O_2_. Protein–protein interactivity in the plasma membrane of *Capsicum annuum* DHN genes, *CaDHN3* and *CaHIRD11*, indicated tolerance towards salt and drought stresses [152]. A wide range of studies have also indicated that DHNs preserve the activities of lactate dehydrogenase (LDH) and malate dehydrogenase (MDH) under freezing and thawing stress damages [102,180].

Group II LEA proteins also aid in the stabilization of cell structures and organelles for preventing the loss of water molecules, as its amphipathic α-helix serves in binding with other biomolecules under conditions of stress and results in the stabilization of said biomolecules [180,181]. Such stabilization of cell structures and organelles was evident through the overexpression of DHN genes in transgenic tomatoes, which improved the relative water content (RWC) and lowered the rate of water loss in the tomatoes [93]. An identical pattern of cell structure stabilization was recognized in group II LEA genes from *Vitis vinifera* and *Rhododendron catawbiense, DHN1a* and *DHN5*. Cell structure stabilization was assigned as an important function of the Φ and K-segments of group II LEA proteins in plants’ reaction to abiotic stresses of dehydration and freezing [44]. Contemporary studies have also revealed the formation of homo- and heterodimeric complexes that bind and provide protection to biomolecules, which in turn protects the structure of cells and organelles and maintains regular cell processes under stress conditions in plants [96].

### 9.2. Scavenging Reactive Oxygen Species (ROS)

Some of the major signaling molecules in plant hormone response pathway are ROS [76]. In terms of ROS, H_2_O_2_ is a versatile molecule that is involved in plants’ reactions to environmental stress [182]. Cells become injured under high concentrations of H_2_O_2_ [183]. Group II LEA genes have a role in lowering the concentration of H_2_O_2_, which lessens the injury to the cells under the stress [6].

**Figure 4:**
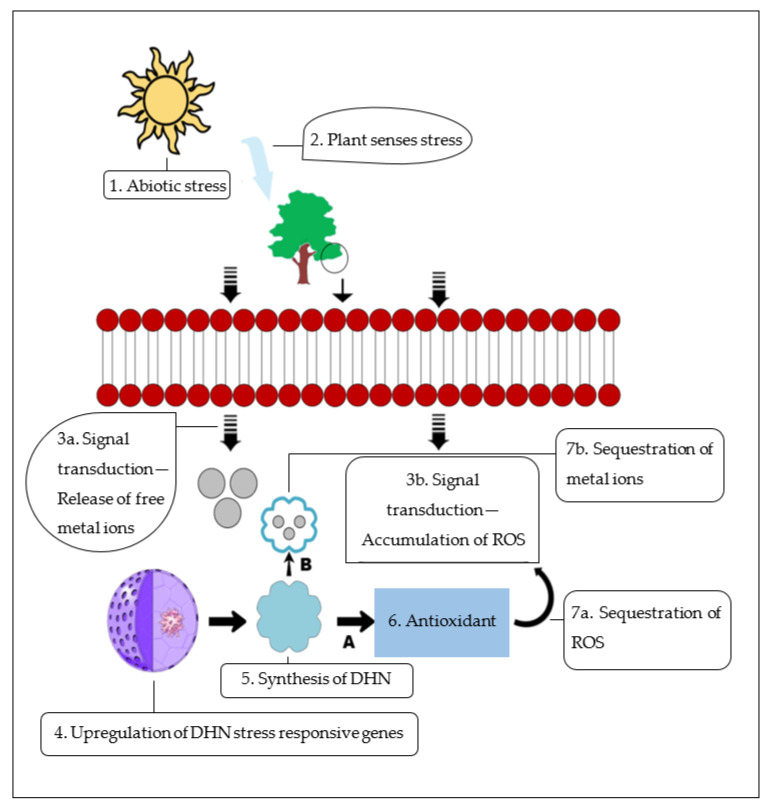
Functional role of DHNs under the abiotic stress (**A**—ROS scavenging as antioxidant, **B**—Metal-ion binding). Under the abiotic stress condition, the plant senses the stress and signals its organelles for the presence of stress through the release of free metal ions or through the accumulation of ROS. After the signal transduction, DHN genes are upregulated within the nucleus, and DHNs are synthesized for the stress tolerance mechanism. Both phenomena (**A**,**B**) can occur within plant cells based on the signal transduction pathway. (**A**) DHNs act as antioxidants and scavenge ROS that accumulate within the plant cells. (**B**) DHNs, through their property of metal-ion binding, also scavenge the free metal-ion radicals that arise within plant cells under abiotic stress.

There are certain group II LEA proteins with the capability of metal binding, which enables them to function as ROS scavengers by removing free radicals under stress [6]. It was demonstrated that the CuCOR19 DHN from *Citrus unshiu*, a K3S type, prevented in vitro peroxidation of liposomes and improved the cold tolerance of transgenic tobacco plants [184]. It was found that an *Arabidopsis thaliana* KS type group II LEA protein, AtHIRD11, lowered the production of ROS from copper metals [185]. However, in KS type DHNs, the extent of the peptides and the contents of histidine influenced the ROS reduction [185]. It has been postulated that DHNs may act as antioxidants [136], which can directly scavenge free radicals (Figure 4A). This radical scavenging activity was suggested to be a result of the high content of amino acid residues susceptible to oxidative modification, such as glycine (Gly), histidine (His), and lysine (Lys), that were targets for radical-mediated oxidation in proteins [184].

### 9.3. Metal-Ion-Binding Protein

DHNs function through their metal-ion-binding properties under certain environmental stresses [10]. The catalytic metal ions, copper and zinc, mainly occur as complexes of metal and protein molecules in plants growing under favorable habitats [100]. However, as plants move under stress conditions, these metal ions can be released as free ions. These ions are involved in ROS production through the Haber–Weiss reaction [178].

Metal ions are a common target for a number of DHNs [6]. Abiotic stresses, such as water stress, result in the release of metal ions from the membranes and organelles and increase the concentration of free metals in the intracellular spaces [112]. It has been hypothesized that DHNs bind to these free metal ions and decrease the damage they cause (Figure 4B) [96].

The binding of DHNs to metal ions has been reported in *Arabidopsis thaliana* and citrus DHNs, AtHIRD11 and CuCOR15, which are able to bind to iron and cobalt over magnesium and calcium and prevent the release of free ions [36]. It has also been found that CuCOR15 acts as a radical scavenger that reduces the metal toxicity in plants under drought stress [36]. Moreover, an ion transport protein (ITP), KS-DHN, from *Ricinus communis* was indicated as an active transporter of metal ions within plants [186].

### 9.4. Phospholipid-Binding Protein

DHNs tend to bind to phospholipids because of their rich K-segments and histidine motifs [10]. Their binding to phospholipids triggers the accumulation of a crucial stress-signaling phospholipid, phosphatidic acid (PA) [96]. The concentration of PA in an inflated plasma membrane is very low, about 1%, but increases under drought stress [41]. The increase in PA concentration is due to low water content within cells or release of ABA [41].

**Figure 5:**
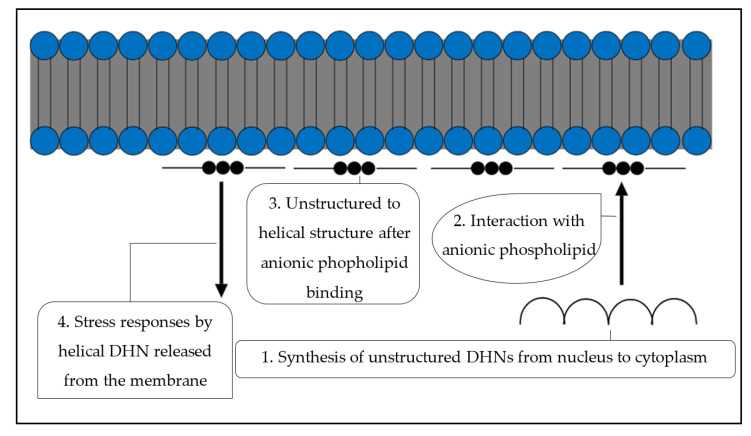
Binding of DHNs to membrane phospholipids. The unstructured DHNs that are synthesized during an abiotic stress in the cytoplasm move close to the cell membranes. Through their phospholipid binding property, the unstructured DHNs bind to the membrane’s anionic phospholipids, attain a helical structure, and generate stress responses. The stress responses include structured DHNs that bind to other stress-sensitive protein molecules and protect them from the damage caused by the stress.

The presence of basic amino acids such as arginine and lysine in DHNs enables them to bind to anionic phospholipids [43]. The interaction between dehydrins and membranes changes certain membrane properties, such as water content and temperature within cells [187]. DHNs bind to charged lipids by the occurrence of electrostatic interactions [188]. Some DHNs gain their helicity structure through binding with acidic phospholipids [180]. This enables them to bind to other biomolecules within the cytoplasm and protect them from stress (Figure 5) [181]. As DHNs bind particularly to acidic phospholipids, it can be postulated that DHNs may interact with membranes of the cell at specific regions [43].

It was shown that a maize SK2-type DHN, DHN1, was able to bind to phosphatidic acid [43]. It has been reported that DHN LT130 from Arabidopsis possessed K-segments with flanking histidine residues that could be regulated by phosphorylation within specific positions at the K-segments; this regulation was assumed to play a key role in lipid vesicle binding [188]. There was immunodetection of acidic DHNs, wheat WCOR414 [19] and Arabidopsis LT129 [189], around the plasma membrane during cold stress, and maize DHNs were found bound to membranes with protein and lipid bodies [190]. The expression of DHNs indicates their functional role under various plant stresses, which necessitates the further examination of the functional processes to strengthen the existing evidence and to identify the potential of group II LEA proteins in the physiological processes of plants that are under environmental stresses.

## 10. Conclusions and Future Perspectives

Environmental and nonenvironmental stresses constantly affect the production of crops. The frequency of both biotic and abiotic stresses is anticipated to increase at a drastic rate. Thus, it is crucial to suit underlying molecular mechanisms and cellular processes that best describe the interrelation between stress-related genes and different stresses. LEA proteins are a remarkably diverse group of proteins with distinct motifs that are involved in plant stress-related responses. Group II LEA proteins, or DHNs, are a highly abundant group of LEA proteins characterized by their high hydrophilicity. DHNs accumulate during seed desiccation and under plant stress conditions, during which they act as functional biomolecules for protecting cells from the damage caused by various abiotic stresses.

The present review reports some investigations on the distribution and differential structural architecture of group II LEA proteins, as well as the molecular expression and regulation of group II LEA genes under various biotic and abiotic stresses, and described the heterologous functional properties of group II LEA proteins. The overexpression of group II LEA genes aided plants in relation to drought, temperature changes, salinity, and osmotic stresses as well as biotic stresses. Group II LEA proteins were distributed in nearly all vegetative tissues under the plant stress condition and during different developmental stages, which indicated their essential property of protecting plants throughout their growth cycle. Group II LEA proteins exhibited a myriad of functions under the different stresses, such as protecting biomolecules and enzymes, radical scavenging, and phospholipid and ion binding. The present review further elaborated group II LEA proteins in *Phoenix dacrylifera* and provided insight to their feasible role in the mechanisms associated with *Phoenix dacrylifera*’s adaptation to its environmental condition. Moreover, in orthodox seeds, various enzymes, proteins, and other transcription factors are desiccation sensitive but protected by DHNs during seed maturation.

The studies on the evolution of group II LEA genes were mainly focused on single species. Examining the evolution of group II LEA proteins as a whole can provide larger insight into their origin and function in plants. Furthermore, group II LEA proteins’ functional properties were mainly analyzed through in vitro experiments, but such experiments are not adequate to substantiate the physiological functions of group II LEA proteins, as most in vitro experiments do not convey results in vivo. Therefore, in vivo studies are essential to exhibit new strategies for effectively breeding stress-tolerant plants using group II LEA genes. Investigation of the morphological changes within plants in response to group II LEA gene expression under abiotic stress conditions will also be required to stipulate their function in stress tolerance via plant morphological changes. As few studies have been undertaken on understanding the functions of DHN against biotic stresses, further studies on group II LEA gene expression in relation to plant hormone and morphological changes exposed to biotic stresses will be essential to identify plants’ stress tolerance mechanisms against biotic stresses and indicate the tangible properties of this versatile protein under various stress conditions. On the whole, group II LEA proteins are effective proteins that can be completely exploited to combat stress and lay a foundation for developing stress-tolerant plants with increased production.

## Figures and Tables

**Table 1:** An overview of recent advancements in knowledge of DHN gene functioning against various abiotic stresses.

S No.	Source	Gene	Year	Target	Tolerance	Reference
1.	Rice	* Rab16A *	2012	Rice	Salinity	[126]
	* Medicago Truncatula *	* MtCAS31 *		Arabidopsis	Drought	[127]
	* Physcomitrella patens *	*PpDHNA* and *PpDHNB*		Arabidopsis	Salt and osmotic	[114]
	Tomato	* tas14 *		Tomato	Drought and salinity	[128]
	Rice	* OsLEA3-2 *		Arabidopsis and rice	Drought and salinity	[129]
	* Opuntia streptacantha *	* OpsDHN1 *		Arabidopsis	Freezing	[130]
2.	* Cerastium arcticum *	* CaDHN *	2013	Saccharomyces	Salinity and freezing	[131]
	* Tamarix androssowii *	* TaLEA *		Poplar	Drought and salinity	[132]
	* Ammopiptanthus mongolicus *	* AmDHN *		Arabidopsis	Drought and salinity	[133]
	* Gentiana triflora *	*GtDHN1* and *GtDHN2*		* Gentiana trifloral *	Drought and freezing	[134]
3.	* Stipa purpurea *	* SpDHN1 *	2014	Arabidopsis	Drought	[135]
	Rice	* OsDhn1 *		Rice	Drought and salinity	[136]
	Arabidopsis	* AtLEA14 *		Arabidopsis and yeast	Salinity	[137]
	* Saussurea involucrata *	* SiDhn2 *		Tobacco	Freezing and drought	[94]
4.	Wheat	* DHN-5 *	2015	Arabidopsis	Salinity	[138]
	* Pennisetum glaucum *	* PgDHN *		*E. coli* and yeast	Salt, osmotic, and heat	[118]
	* Solanum habrochaites *	* ShDHN *		Tomato	Cold, drought, salt, and osmotic	[93]
	* Olea europaea *	* OesDHN *		Arabidopsis	Drought	[139]
5.	* Vitis vinifera *	* VvDhn *	2016	Tobacco	Drought and salinity	[140]
	Wheat	* DHN-5 *		Arabidopsis	Salt and osmotic	[119]
	* Eucalyptus nitens *	* EniDHN2 *		Arabidopsis	Cold	[141]
	Wheat	*TaDHN1* and *TaDHN3*		Arabidopsis	Drought and salinity	[104]
6.	* Prunus mume *	* PmLEAs *	2017	Tobacco	Drought and cold	[107]
	* Hevea brasiliensis *	*HbDHN1* and *HbDHN2*		Arabidopsis	Salt, drought, and osmotic	[79]
	* Saussurea involucrata *	* SiDHN *		Tobacco	Drought and cold	[142]
7.	Bermudagrass	* CdDHN4 *	2018	Arabidopsis and *E. coli*	Salinity, cold, and heat	[143]
	* Ipomoea pes-caprae *	* IpDHN *		Arabidopsis	Salt and drought	[81]
	* Eriobotrya japonica *	* EjDHN *		Tobacco	Cold	[144]
	* Gastrodia elata *	* GeLEA *		* E. coli *	Cold	[145]
8.	* Malus domestica *	* MdoDHN11 *	2019	Arabidopsis	Drought	[24]
	* Oryza sativa *	* OsDhnRab16 *		Rice	Drought	[146]
	* Capsicum annuum *	* CaDHN5 *		Arabidopsis	Salt and osmotic	[125]
	Korshinsk pea shrub	* CkLEA2-3 *		Arabidopsis	Salt and osmotic	[120]
	African lily	*ApY2SK2* and *ApSK3*		Arabidopsis	Salt, osmotic, cold, and drought	[121]
	*Zea mays*	* ZmDHN13 *		Yeast and tobacco	Oxidative stress	[147]
9.	* Ammopiptanthus mongolicus *	*AmDHN132*, *AmDHN154* and *AmDHN200*	2020	Arabidopsis	Salt, osmotic, and cold	[123]
	* Cerastium arcticum *	* CaDHN *		Arabidopsis and *E. coli*	Salt, cold, and drought	[148]
	* Medicago falcate *	* MfLEA3 *		Tobacco	Cold and drought	[149]
	* Capsicum annuum *	* CaDHN4 *		* Capsicum annuum *	Salt	[77]
	*Cucumis melo*	* CmLEA-S *		Tobacco	Salinity and drought	[150]

10.	* Vitis vinifera *	*VviDHN2* and *VviDHN4*	2021	* E. coli *	Freezing and drought	[151]
	* Capsicum annuum *	*CaDHN3* and *CaHIRD11*		Arabidopsis	Salt and drought	[152]
